# Functional comparison of pacifiers using finite element analysis

**DOI:** 10.1186/s12903-022-02087-4

**Published:** 2022-03-02

**Authors:** David A. Tesini, Linda C. Hu, Brent H. Usui, Christopher L. Lee

**Affiliations:** 1 Department of Pediatric Dentistry, Tufts University School of Dental Medicine, 1 Kneeland St., Boston, MA 02111 USA; 2 Olin College of Engineering, 1000 Olin Way, Needham, MA 02492 USA

**Keywords:** Pacifier, Palate, Finite element analysis, Non-nutritive sucking, Malocclusion, Posterior crossbite

## Abstract

**Background:**

Pacifiers have been shown to affect maxillary growth related to the anatomic structure of the palate and forces placed upon it during sucking. This study compares and evaluates the mechanical behavior of pacifiers of different design and size (*i.e*., fit), identified by brand and size, positioned in age-specific palatal models with respect to both contact area and force when subjected to peristaltic tongue function and intraoral pressure related to non-nutritive sucking.

**Methods:**

Nonlinear finite element analyses were used to simulate dynamic mechanical interaction between the pacifiers and palates. Time-varying, external pressure loads were applied which represent intraoral pressure arising from non-nutritive sucking and peristaltic behavior of the tongue. The silicone rubber pacifier bulb was represented using a hyperelastic material model.

**Results:**

Results from the finite element analyses include deformation, stress, strain, contact area, and contact force. Mechanical interaction was evaluated in terms of the spatial distribution of the contact area and force between the pacifier and the palate. The resulting palatal interaction profiles were quantitatively compared to assess how pacifier fit specifically affects the support provided to two areas of the palate, the palatal vault and the Tektal wall.

**Conclusions:**

Pacifiers interact with the palate differently based on their fit (*i.e.*, design and size) regardless of whether they are labeled conventional or orthodontic. Finite element analysis is an effective tool for evaluating how a pacifier’s design affects functional mechanics and for providing guidance on biometric sizing.

## Background

The worldwide use of pacifiers by infants and toddlers is well known. Soothing, comfort, pain relief, sucking and feeding skill development, coordination of sucking reflexes, and prevention of SIDS (sudden infant death syndrome) are some of the reasons given for the increasing prevalence of pacifier use. However, the mechanical behavior of pacifiers, especially interaction within the palate, is not well characterized quantitatively. Non-nutritive sucking (NNS) behaviors are known to cause adverse dental and oral myofunctional effects in children [1, 2]. Specific effects on the growth and anatomic changes of the palate result from duration and frequency of use, infant sucking dynamics, pacifier fit, and functional tongue movement in an intraoral environment of negative pressure. These are the predictors for developing malocclusions of anterior open bite and posterior crossbite from birth to 48 months [3]. The most serious persistent orthodontic effect of pacifier use is the development of the posterior unilateral functional crossbite [4], as the anterior open bite has been shown to often be self-correcting [5].

Many factors come into play during morphological development of the palate, and the forces of NNS have a direct effect on the structures of the palate [6–8]. These forces acting on the palatal suture, palatal shelves, Tektal plates, and the palatal vault contribute to alveolar and palatal grooving, high arch palates, palatal constriction, dental crossbites, compromised airways, facial asymmetries, and changes in the form and dimension of the palate [6–11].

In particular, the median palatal suture, a fibrous articulation at birth, is not fused and the transverse suture between the palatine process of the maxilla and intermaxillary bone is open. The palate is relatively high arched; and the soft bones of the palate and the lateral palatine processes are malleable [9, 12]. Palatal growth responds to forces of the tongue, the peristaltic movement of the tongue, intraoral pressure, position of bulb placement in the oral cavity, composition and design characteristics of the pacifier bulb, and the design of the pacifier shield. Sucking requires control of many oral-myo-functions which occur concomitantly with increasing and decreasing pressures of a sucking cycle [13, 14]. Transverse changes in palatal anatomy have been shown to be affected by tongue pressure on the hard palate [15].

Design characteristics of conventional, non-orthodontic (*e.g.*, cylindrical and cherry) and orthodontic (physiologic shape) pacifiers, are based on unreported metrics and vary from brand to brand. Recommendations on staging of pacifier size, *i.e*., based on chronological age, cause confusion for parents attempting to choose the right size for their baby or infant. The *fit* (*i.e*., design and size) is important because it determines the (1) position and seating of the pacifier bulb in the palate, (2) functionality of the pacifier bulb design related to claims made on packaging and in advertising, (3) contact of the pacifier shield with the face, and (4) comfort and soothe-ability of the pacifier.

The designs of pacifier bulbs vary in dimension, shape, and material properties. Levrini et al. [16] first studied pacifiers with different geometry using finite element analysis (FEA). Although this early study did not consider peristaltic behavior and intraoral pressure, it did demonstrate the importance of contact between the pacifier and the palate. They reported on the impact of a pacifier’s design to support the palate against collapse caused by the inward pressure of the buccinator muscles on the maxillary arch and to preserve the transverse dimension. Using FEA, Freitas [17] evaluated the distribution of mechanical stresses and displacements on the palate from contact with three different pacifier geometries in three-year-old children. Recently, Maurya et al*.* [18] conducted an FEA study to evaluate the mechanical behavior of orthodontic and conventional pacifiers in comparison to a human nipple model.

In distinct contrast to the three FEA studies above, Lee et al*.* [19] performed dynamic, large-displacement analyses with hyperelastic materials which captured the mechanical interaction between the pacifier and the palate and calculated resulting stresses, strains, deformation, contact areas, and contact forces/pressures. Palatal interaction due to pacifier distortion from peristalsis and intraoral pressure during NNS was quantitatively characterized and evaluated in their analysis process. Alternatively, another study [20] reported on an image-processing technique based on anthropometric and physical parameter correlations to describe and evaluate interaction of a pacifier with the palate.

Recent litigation in the USA against a pacifier manufacturer will likely lead to a need for greater understanding of how design, size, and mechanical behavior relate to the causes of dental malocclusions [21]. Pacifier manufacturers are now being asked for validation of packaging claims made to the consumer in an age where quantifiable biometric parameters are widely available. As such, finite element analysis would find ready application.

The aim of this study is to use the dynamic, nonlinear finite element analysis process of Lee et al*.* to evaluate the mechanical behavior of conventional and orthodontic pacifiers (identified by brand and size) positioned in age-specific palatal models with respect to both contact area and force when subjected to tongue function and intraoral pressure related to NNS.

## Methods

Finite element simulations were used to study mechanical interaction between pacifiers of different types and sizes, and palates of different ages.

### CAD models

**Fig. 1:**
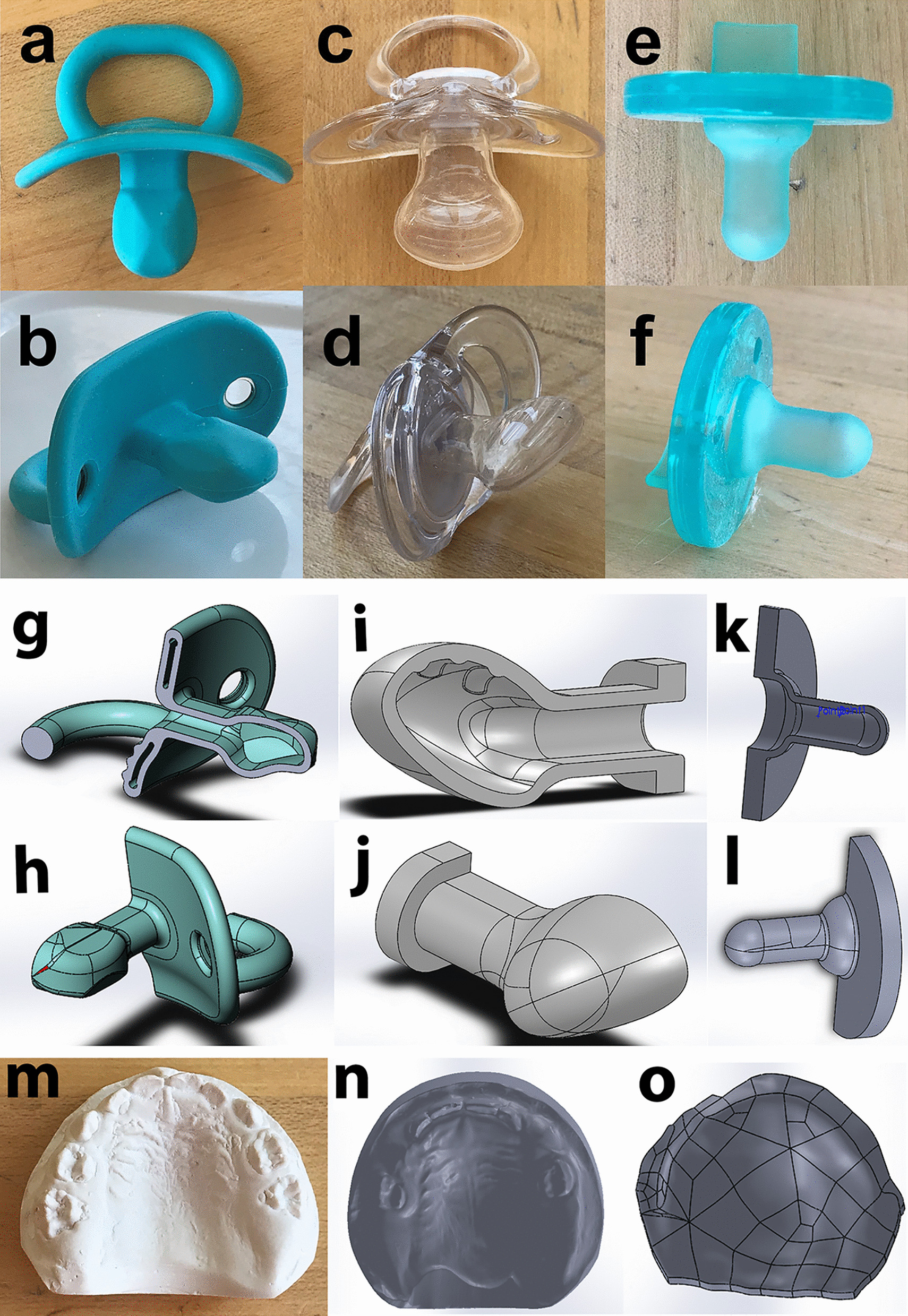
Example pacifiers: **a**, **b** Tomy Boon (3 month +); **c**, **d** tommee tippee (6–18 months); and **e**, **f** Soothie (0–3 months). Example pacifier CAD models, symmetric one-half model section views with respect to the median plane: **g**, **h**, Tomy Boon (3 month +); **i**, **j**, tommee tippee (6–18 months); and k-l, Soothie (0–3 months). Example palates: m, plaster cast from a 22-month-old child; n, scanned image of a 12-month cast; and o, CAD model of 0-month palate

Finite element models were created for the commercial pacifiers (see examples in Fig. 1a–f) listed in Table 1 based on CAD (computer-aided-design) solid models (see examples in Fig. 1g-i) generated in SolidWorks (version 2019, Dassault Systèmes SE, Waltham, MA). The pacifiers were designated by name and size based on the maximum (horizontal) width of the bulb and the recommended age range for use by the manufacturer.

**Table 1:** Pacifiers’ size and recommended age for use

Size	S (non-ortho.)		TT (ortho.)		TB (ortho.)	
	Width	Age	Width	Age	Width	Age
1	12.5	0–3	17.0	0–3	16.3	0–3
2	14.0	3+	20.0	3–6	17.8	3–9
3	–	–	24.1	6–18	20.0	9+

S: Conventional, non-orthodontic bulb based on Soothie (Philips Advent, Glemsford, Suffolk, England) design

TT: Closer to Nature, 2007–2014 version (tommee tippee, Mayborn USA Inc., Stamford, CT). Orthodontic

TB: Jewl (Boon, Tomy International, Inc., Oak Brook, IL). Orthodontic. The design of the bulb also changes with size

Size: designation used for this study

Width: maximum bulb width (mm)

Age: manufacturers’ recommended age range for use (months) at the time of this study

CAD solid models of three palates of ages 0, 12, and 22 months were generated from three-dimensional scans of plaster casts; see examples of each in Fig. 1m–o. The CAD models for the pacifier bulbs and palates were combined in SolidWorks as assemblies. The Tektal wall and palatal vault area delineations were defined by a groove system described and modeled by Hohoff et al*.* [9].

### Finite element models

**Fig. 2:**
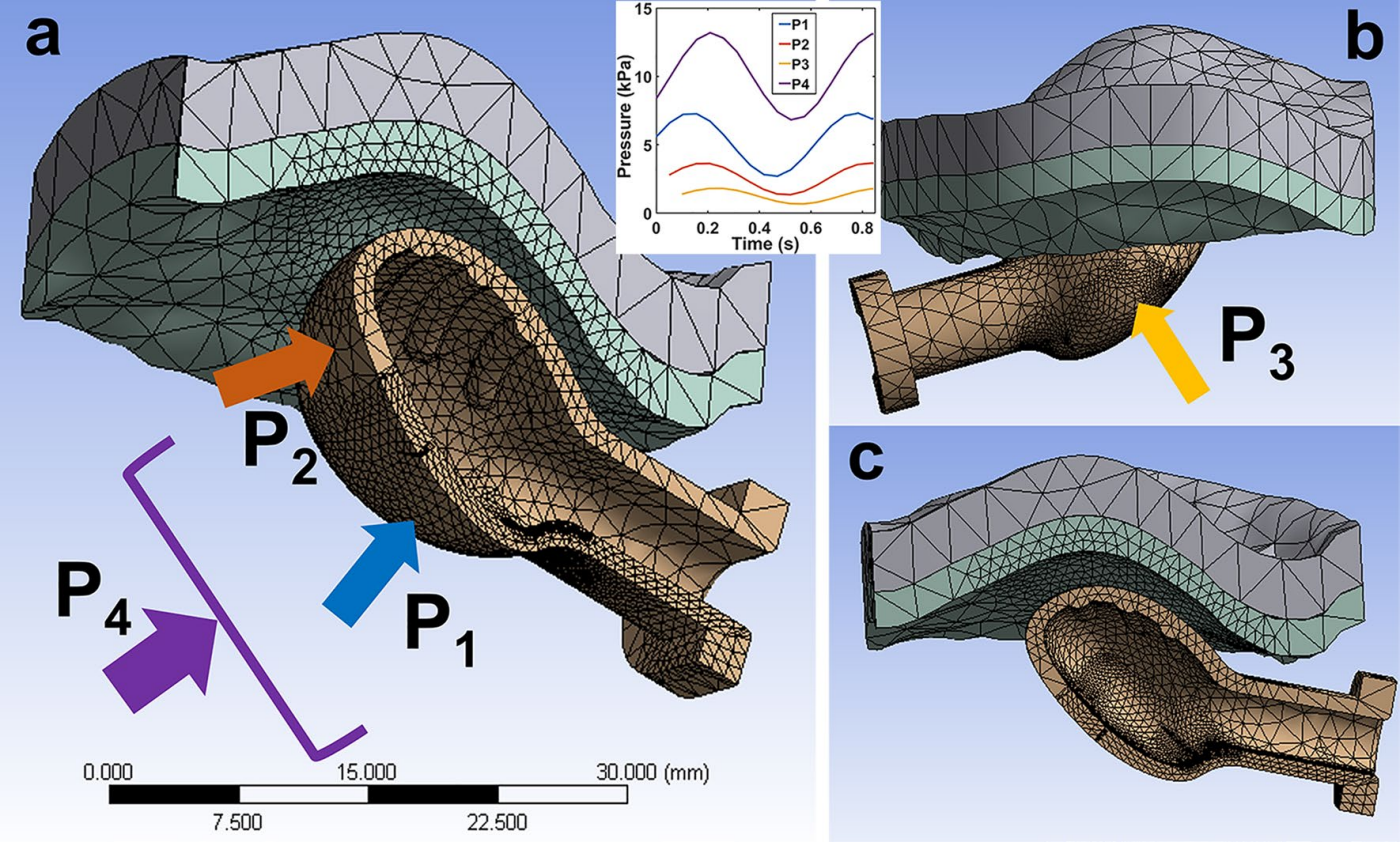
Finite element model of the size 2 TT pacifier and 12-month-old palate. One-half symmetry model of the undeformed mesh, **a** isometric view facing the median plane; **b** lateral side view; and **c** medial side view. Two layers of elements of the palate: mucosa (inferior, green) and palatine process of maxilla (superior, gray). Dynamic pressure loads are applied to free surfaces around the locations indicated by (colored) arrows. Peristaltic motion of the tongue: P1, P2, and P3. Intraoral non-nutritive sucking pressure: P4. Pressure load time histories (graph insert) over 1.3 non-nutritive sucking cycles (1.56 Hz frequency, 0.64 s period). Time delays of 0.05 s (P2) and 0.1 s (P3) represent staggered contact as the tongue engulfs the pacifier bulb

Finite elements models were generated from the CAD assemblies as curvature-based meshes with increased refinement near the end of the bulb to minimize element distortion. As the median plane is a plane of geometric symmetry, the finite element models were taken to be one half of the CAD model with respect to the median plane. An example finite element model of the TT size 2 pacifier bulb with the 12-month-old palate is shown in Fig. 2. The mesh is made up of 59,123 nodes and 37,745 solid elements (10-node, second-order tetrahedrons).

### Material models

The pacifier bulbs were represented as medical-grade silicone rubber (MED 4950) with density, ρ = 1140 kg/m^3^, using a hyperelastic, five-parameter Mooney-Rivlin model with parameters C10 = 87,833 Pa, C01 = -20,313 Pa, C20 = 14,668 Pa, C11 = -202.93 Pa, and C02 = 16.386 Pa, and incompressibility factor, D1 = 1e-6 Pa^−1^ [22].

The palate was represented by two bonded (no relative motion) layers of solid elements. On the inferior side of the palate is a mucosa layer (nominal thickness 3 mm) represented as a linear elastic material with parameters, density, ρ = 1100 kg/m^3^, Young’s (elastic) modulus, E = 1.0e6 Pa, Poisson’s ratio, ν = 0.3, and, bulk modulus, β = 8.33e5 Pa [23]. The superior side of the palate was represented as a linear elastic material (nominal thickness 5 mm) with parameter values from metrology measurements from palatine process of maxilla, density, ρ = 3880 kg/m^3^, Young’s (elastic) modulus, E = 1.41e10 Pa, Poisson’s ratio, ν = 0.3, bulk modulus, β = 1.18e10 Pa [24].

### Finite element analysis

In the simulations, the pacifier bulbs were constrained (fixed-no translation or rotation) at its base and the palate was fixed on the superior surface. Initially the bulb was not in contact with the palate. The base (shield-side) of the bulb is 5 mm from the most anterior point of the palate and oriented at an angle of 5° with respect to vertical. In some cases, this angle was increased up to 10° to address numerical convergence issues. The superior surface of the bulb and the mucosa layer were defined as frictionless contact surfaces. The inside wall of the pacifier bulb was defined as a single contact surface. Under large pressure on its outside surface, the bulb can collapse allowing the interior of the bulb to come into contact with, but not penetrate, itself.

Four different dynamic, external pressure loads were applied to the finite element models to represent the intraoral pressure due to non-nutritive sucking (NNS) and the peristaltic behavior of the tongue; see Fig. 2 inset. The pressure loads are sinusoidal with a frequency of 1.56 Hz (period = 0.64 s). The first load (P1) is the contact pressure (amplitude = 4610 Pa) from the tongue on the inferior surface of the bulb. The second (P2) is the contact pressure (amplitude = 2305 Pa) from the tongue as it wraps around the bottom of the end of the bulb. The third (P3) is the contact pressure (amplitude = 1140 Pa) from the tongue as it cups the sides of the bulb. The fourth load (P4) is the intraoral, NNS pressure (amplitude = 6370 Pa) applied to the free surface on the anterior side of the bulb. Short time delays of 0.05 s and 0.1 s were included in the second and third tongue-contact pressure loads, respectively, to capture the temporal progression of the tongue engulfing the bulb. This loading is based on measurements reported in [25].

All of the simulations are nonlinear, large-displacement, transient dynamic analyses performed using ANSYS Workbench (ver. 18.2, Ansys Inc., Canonsburg, PA) which were run for 1.3 NNS cycles (2.15 s). Pressure loads were linearly ramped up from zero to their initial values to start the loading cycles.

## Results

**Fig. 3:**
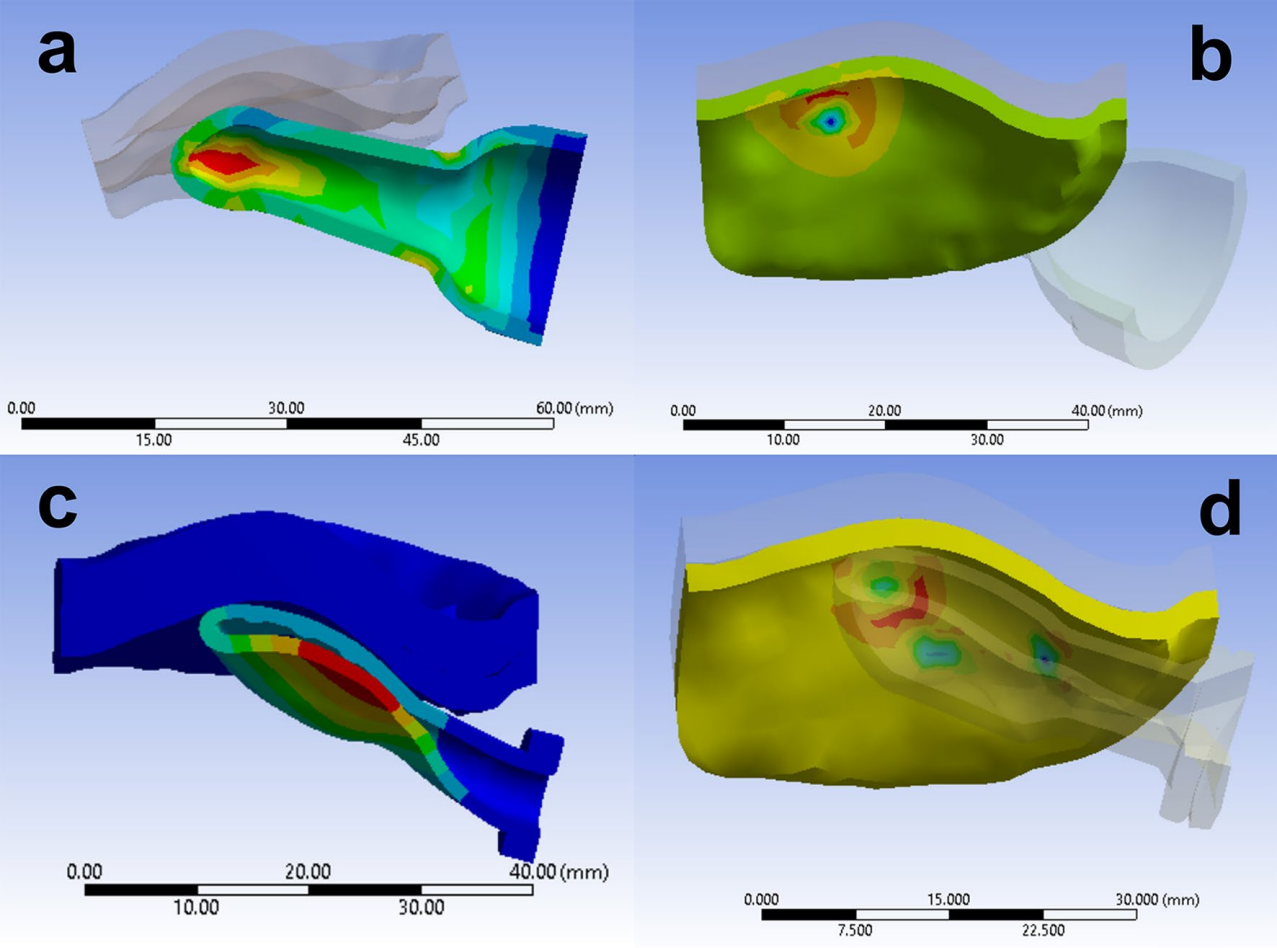
Sample FEA results. Contours superimposed on deformed pacifier and 12-month-old palate, **a** size 3 S pacifier, equivalent plastic strain, maximum value 0.150, t = 1.48 s (palate is translucent), **b** size 3 S pacifier (translucent), maximum principal stress, maximum value 1.06e4 Pa, t = 2.15 s; **c** size 3 TT pacifier, total deformation, maximum value 11.87 mm, t = 1.06 s, and **d** size 3 TB pacifier (translucent), maximum principal stress, maximum value 7.68e4 Pa, t = 2.15 s

Results from FEA simulations include stress, strain, displacement, contact area, and contact force/pressure. For this study, simulations were run over a specified time range so results are available and can be plotted as functions of time. Figure 3 shows some example results. Equivalent elastic strain (Fig. 3a), maximum principal stress (Fig. 3b, d), and total deformation (Fig. 3c) are shown as color contours superimposed on their corresponding FEA model in the deformed state at a given time.

**Fig. 4:**
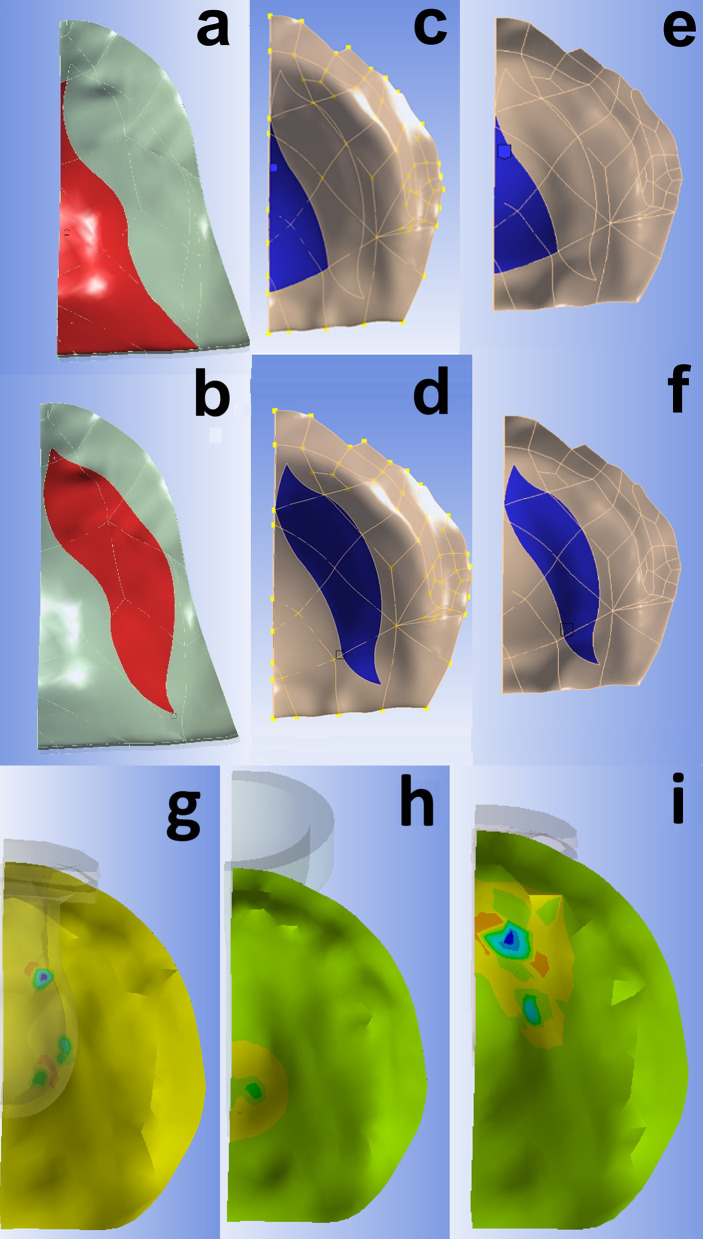
Palatal areas on finite element models. **a**, **c**, **e** palatal vault area (in color) of 0, 12, and 22-month palates, respectively. **b**, **d**, **f** Tektal wall areas of 0, 12, and 22-month palates, respectively. Example contact area contours of maximum principal stress on 12-month palate: g, size 3 TB pacifier (t = 0.79 s)-three areas of contact; h, size 3 S pacifier (t = 0.91 s)-one area of contact; and i, size 1 TB (t = 0.91 s)-one large area of contact in the TW. Pacifiers are translucent

In order to characterize the spatial distribution of the contact between the pacifier and the palate, the inferior surface of the palate was separated into several areas. The two that are of interest herein are the palatal vault (PV) and Tektal wall (TW) which were defined following [9]. In Fig. 4a–f, the PV and TW areas are highlighted in color on the FEA models of the 0, 12, and 22-month palates. The PV is bounded on one side by the median plane and lies towards the posterior. The anterior portion of the TW is adjacent to the median plane then extends bilaterally in the posterior direction.

The FEA results include the total contact area and force of a pacifier bulb with the palate (see examples in Fig. 4g–i) as well as the contact within the PV and TW. In the figures following, comparisons are made between contact in the PV and TW of pacifiers with different designs/sizes and different-aged palates as a function of time over the loading cycle. The dashed (solid) lines correspond to the PV (TW).

**Fig. 5:**
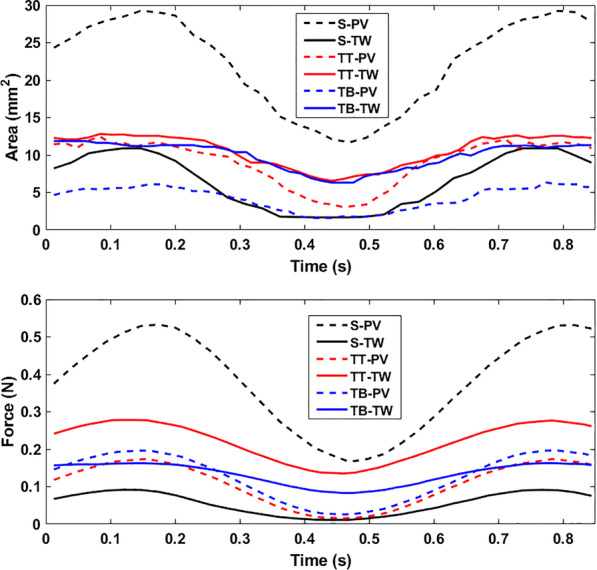
Contact areas and forces for size 1 S, TT, and TB pacifiers in 0-month palate. Time versus contact area (top) and contact force (bottom) over 1.3 non-nutritive sucking cycles. Dashed (solid) lines for palatal vault (Tektal wall). All three pacifiers contact the TW

Figure 5 shows the size of the contact area and force between the size 1 S, TT, and TB pacifiers and the 0-month palate. The non-orthodontic S pacifier has the greatest contact with the PV but the least with the TW. The orthodontic pacifiers, TT and TB, have similar contact with the PV and TW. For this small palate, all the pacifiers support the TW. The orthodontic bulbs (TT, TBA) do so with greater force than the non-orthodontic bulb (S).

**Fig. 6:**
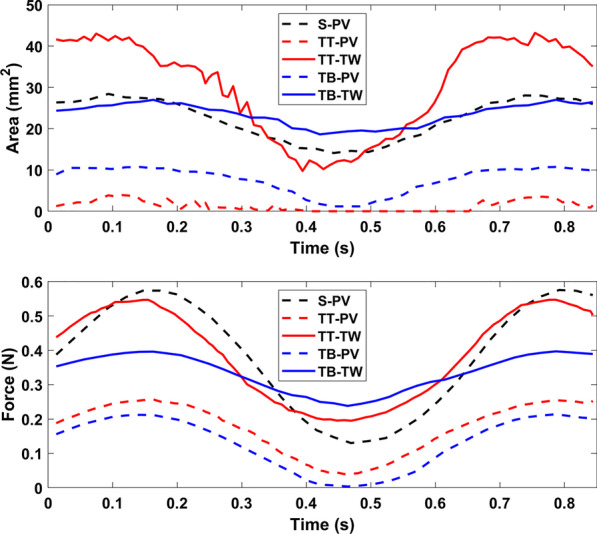
Contact areas and forces for size 2 S, TT, and TB pacifiers in 12-month palate. Time vs. contact area (top) and contact force (bottom) over 1.3 non-nutritive sucking cycles. Dashed (solid) lines for palatal vault (Tektal wall). The S pacifier does not contact the Tektal wall

In Fig. 6, the contact area and force between the size 2 S, TT, and TB pacifiers with the 12-month palate are shown. Because the cylindrical-shaped S pacifier bulb is not as wide as the TT and TB pacifiers, it does not come into contact with the TW. As a result, the S pacifier does not provide support across the width of the palate. The TB pacifier contacts the TW more than the TT pacifier but with less contact force.

**Fig. 7:**
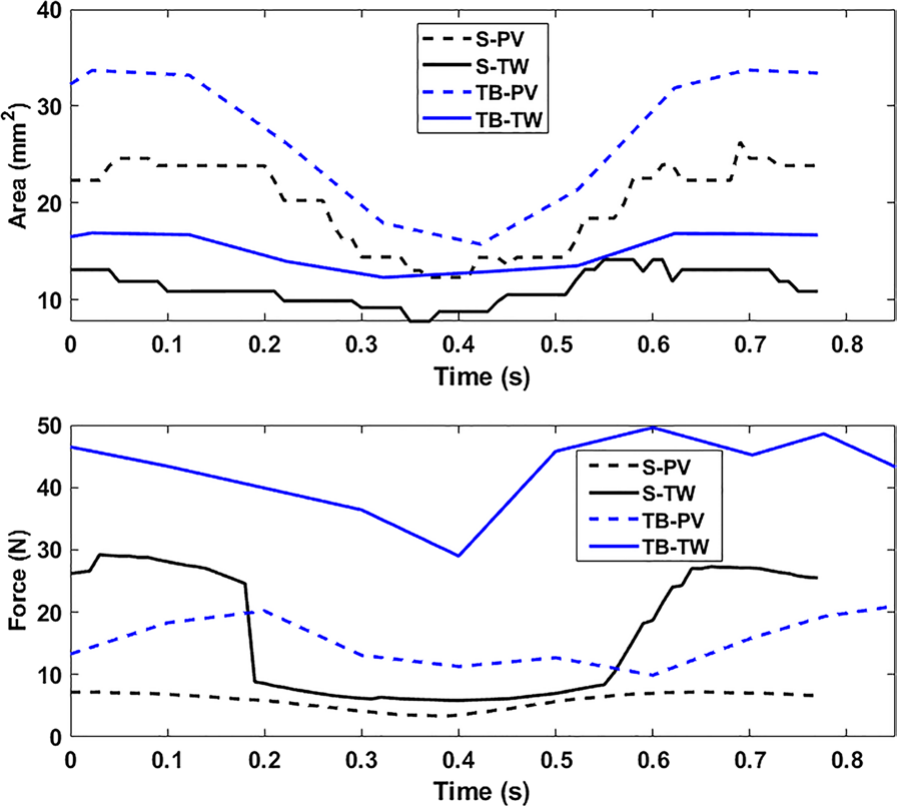
Contact areas and forces for size 2 S and size 3 TB pacifiers in 22-month palate. Time vs. contact area (top) and contact force (bottom) over 1.3 non-nutritive sucking cycles. Dashed (solid) lines for palatal vault (Tektal wall). The TB pacifier comes into greater contact than the S pacifier in both the PV and TW

Figure 7 shows the contact area and force between the size 2 S and the size 3 TB pacifiers with the 22-month palate. The orthodontic pacifier, TB, comes into greater contact than the non-orthodontic pacifier, S, in both the PV and TW. Note that the S pacifier contacts the PV of the 22-month palate but not the 12-month palate.

For the results shown in Figs. 5, 6 and 7 the conventional and orthodontic pacifier bulbs respond differently to peristalsis of tongue and intraoral pressure of NNS. The orthodontic pacifiers provide more support in the Tektal wall area. In order to observe palatal contact area and force for pacifiers that are properly matched with respect to manufacturer’s recommended age, simulations of a given pacifier with different-aged palates were performed. In the following comparisons, normalized contact area is defined as the contact area divided by the total area of the corresponding palate.

**Fig. 8:**
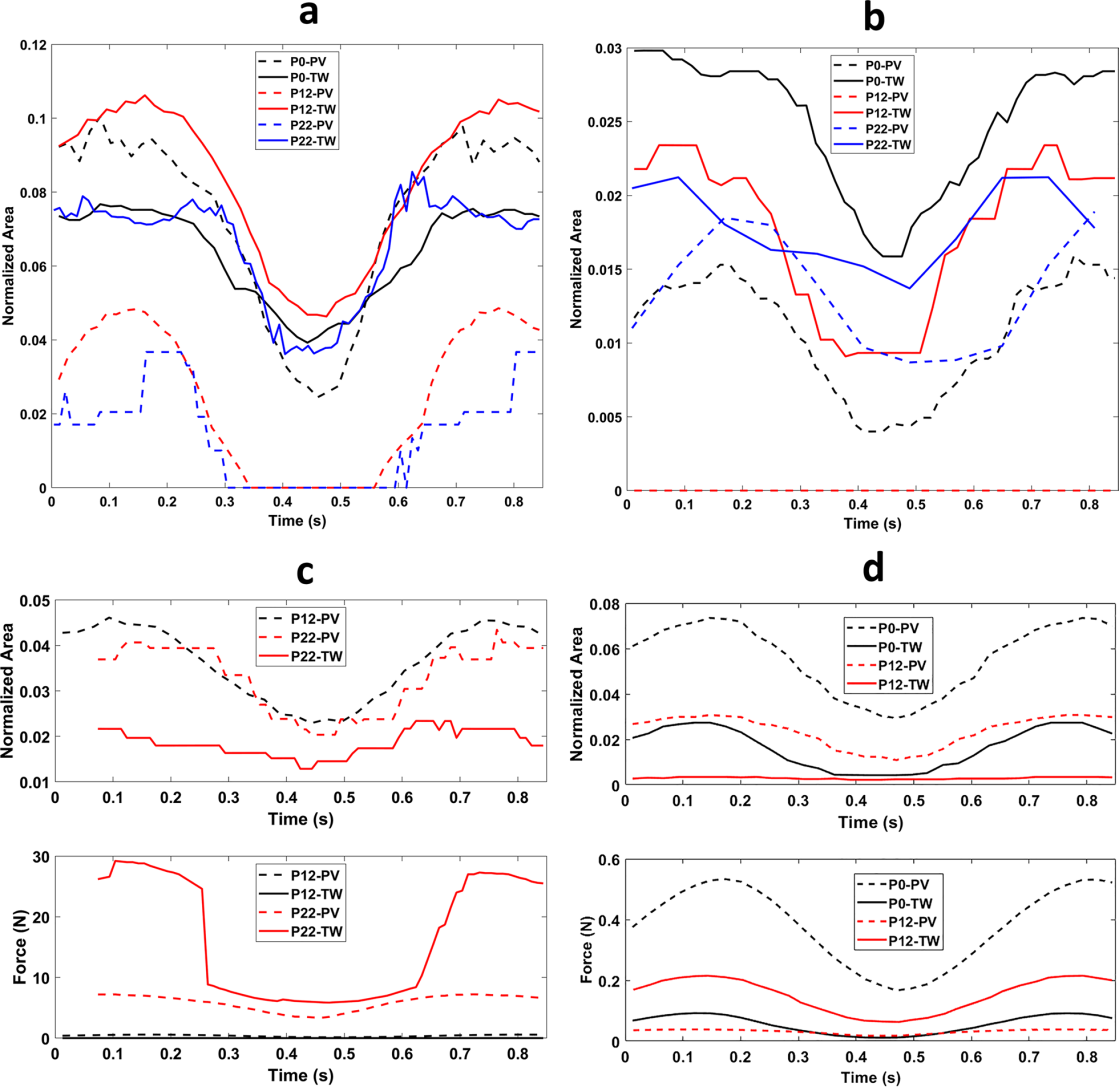
**a** Time versus normalized contact areas for size 1 TT pacifier in 0, 12, and 22-month palates. **b** Time versus normalized contact areas for size 1 TB pacifier in 0, 12, and 22-month palates. **c** Time versus normalized contact areas and forces for size 2 S pacifier in 12 and 22-month palates. **d** Time versus normalized contact areas and forces for the size 1 S pacifier in the 0 and 12-month palates. Dashed (solid) lines for palatal vault (Tektal wall)

In Fig. 8a, the contact area results for the size 1 TT pacifier with the 0, 12, and 22-month palates are shown. The pacifier contacts the TW of all three palates and provides support even when the bulb is undersized. Due to the shape of the bulb, there is more contact in the TW than the PV. Figure 8b is a plot of time vs. contact area for the size 1 TB pacifier with the 0, 12, and 22-month palates. This pacifier contacts the TW of all three palates; even though the pacifier is undersized in the last two cases. Note, the bulb does not contact the PV of the 12-month palate (see Fig. 8c) but does so with the 0 and 22-month palates due to the different initial positions of the bulb with respect to the palate.

In Fig. 8c, the contact area and force between the size 2 S pacifier and the 12 and 22-month palates are shown. The conventional, non-orthodontic bulb comes into greater contact with the PV than the TW of both palates. The bulb does not contact the TW of the 12-month palate because the single contact area is towards the posterior of the palate in the PV. Figure 8d are plots of contact area and force between the size 1 S pacifier and 0 and 12-month palates. The conventional bulb is too small for the 12-month palate in the sense that it has minimal contact with the TW.

## Discussion

This is the first study (based on the authors’ literature review) that applies nonlinear, dynamic finite element analyses to calculate the area and force of palatal contact under peristaltic action of the tongue and intraoral pressure of NNS. The results give new insights into the need to define *pacifier fit* by using the metrics of both size (*e.g*., bulb width) and the design (*e.g.*, geometric, mechanical, physiological) of the individual pacifier.

The tongue and pacifier bulb interact with the palatal vault, Tektal wall (lateral palatal shelves), and anterior and peripheral alveolar pads during NNS. Variations in size and design (conventional or orthodontic) cause different patterns of palatal support against the Tektal wall and the palatal vault.

Commercially available orthodontic pacifiers vary in size, shape, and physiologic design. For example, the TB and TT pacifiers are of different “orthodontic” design but, in general, behave similarly with respect to their palatal interaction. Their mechanical behavior, however, is substantially different from conventional pacifiers such as the Soothie.

The conventional size 1 S pacifier in the 0-month palate shows greater contact and force in the PV area by nature of its design and size in comparison to the TB and TT orthodontic pacifiers. Because the 0-month palate is small, all three pacifiers came into contact with the TW; however, the TT showed the greatest area of contact and force against the Tektal wall.

For the orthodontic size 2 bulbs in a 12-month palate, there is contact on the TW by the TT and TB pacifiers but no contact by the S pacifier. Both size and design have limited this contact. The force is predominantly distributed in the PV. A pacifier that is *too small*, regardless of the design, is one whose bulb does not contact in the TW (lateral palatine area) under peristaltic tongue movement and intraoral pressure.

For the orthodontic size 3 TB bulb in a 22-month palate (Fig. 7) the greatest contact area/force was seen in the TW. Surprisingly there is no contact of this bulb in the PV of the 12-month palate. This is due to the fact that the geometric angled design provided an observable fit profile in both FEA and image-collisions analysis [20]. This observation highlights the need to evaluate the differences in design of one brand’s pacifier to another and shows that orthodontic pacifiers cannot be placed into a single stratified “orthodontic” pacifier grouping.

Most significantly, the fit of the pacifier in different-aged palates shows large differences in palatal contact and force when the same pacifier is evaluated in different-aged palates, as shown in Fig. 8. As growth of the palate progresses, contact area/force profiles change. The loss of any contact in the Tektal wall which favors contact in the palatal vault area may contribute to palatal atresia as seen in palatal grooving caused by oro-tracheal tubes [12]. For example, a size 2 S pacifier in a 12-month palate resulted in no Tektal wall contact. Likewise, the size 1 TT has lost contact with the Tektal wall in the 22-month palate. This supports the concern that chronological age sizing may not present reliable recommendations across all brands.

The difficulty in making comparisons with previous FEA studies is due in part to the fact that size, design, and fit have not been inclusively evaluated using dynamic simulations; constitutive models have been limited to linearly elastic materials; and biometric size of the pacifiers have not been disclosed. In a broad-sense, however, our results concur with Levrini et al. [16] who used a palatal model of a newborn and found that different geometric designs (conventional, orthodontic, and cherry) have different stress–strain contact profiles. In agreement with this study, the pacifier contact area within the palate was shown to vary based on the geometric shape of the pacifier. The pattern of stress distribution can have a direct effect on the morphological development of the palatal structures. Levrini et al. did not assess the fit of the pacifier in the palate but did find that the position of the pacifier in the palate differed from one geometric shape to another. Their results support the premise that the pattern of stress distribution can have a direct effect on the morphological development of the palatal structures.

Freitas’ findings [17] that an orthodontic pacifier produced maxillary force both forward and to the sides toward the lateral supporting pillars concur with this study. Conversely, a conventional, symmetrical cylinder design pacifier (*e.g*., Super Soothie) promoted an upward deformation in the midpalatine suture, favoring development of a more atretic palate. Pacifier bulb dimensions were not reported in their study.

Although Maurya et al*.* [18] used a geometric model of a pacifier and palate, they did not provide data on dimensional fit into aged palates. Their investigation study used “only average dimensions of a human infant,” dentate and edentulos gum pads, and a uniformly applied “biting” pressure [18]. No dimensional information or brand names were given for the pacifiers in their study. It is important to reiterate that previous FEA studies [16–18] were all limited to static loading and linear elastic materials.

It is recognized that results of comparison studies will vary based on initial positioning of the bulb with respect to the palate, the shape of the palate, and the nature of the loading (*e.g*., amplitude, frequency, and direction). For the conditions and loading used in this study, comparison of results relative to each other show that the orthodontic pacifiers provided more support in the TW area than the conventional, non-orthodontic pacifiers. It should also be noted that the palatal models used were representative of the age but there are normal deviations in each age group.

The findings of this study show that the size metrics, geometric, mechanical, and physiological design of the pacifier alter the functioning and mechanical behavior of pacifiers during NNS. Any parameter of pacifier fit that can contribute to the loss to transverse dimension or cause palatal atresia can lead to the development of posterior crossbites and other malocclusions. This is integral to oral facial growth dynamics which impacts not only the development of malocclusions but can also compromise the airway and result in abnormal oral myofunction.

These FEA results show how pacifier fit during growth stages can play a significant role in palatal development. When coupled with duration, frequency, and intensity of use, these results provide new insights into the development of malocclusions arising from NNS.

## Conclusions

In this study, nonlinear finite element analyses are used to evaluate the mechanical interaction (*e.g.,* contact area, contact force, deformation, strain, stress) of conventional and orthodontic pacifiers (identified by brand and size) positioned in age-specific palatal models with respect to both contact area and force when subjected to tongue function and intraoral pressure related to non-nutritive sucking.

The resulting dynamic palatal interaction profiles show that (1) pacifiers behave differently, e.g., amount of contact in the TW region, based on their size and design regardless of whether they are labeled conventional or orthodontic and (2) contact (area and force) in or around the palatal midline only means that the pacifier is either sized too small or there are limitations in its design.

FEA is an effective tool for evaluating how a pacifier’s design affects functional mechanics and for providing guidance on biometric sizing. In the cases analyzed herein, the fit (*i.e.*, design and size) of the pacifier determines the contact (area and force) between the pacifier and palate during peristalsis and under intraoral pressure. Such results contribute to advances in the understanding of how sizing and prolonged pacifier interaction with the palate can affect palatal growth in infants and toddlers. Knowledge of the functional behavior of pacifiers and their mechanical interaction with the palate can be used to provide guidance for the development of Oral Health Policy statements by leading pediatric organizations.

## Data Availability

The material, including CAD and FEA models, that support the findings of this study are available from the corresponding author upon reasonable request.

## Abbreviations

CAD: Computer aided design
FEA: Finite element analysis
NNS: Non-nutritive sucking
PV: Palatal vault
SIDS: Sudden infant death syndrome
TW: Tektal wall
